# Cognitive Impact and Psychophysiological Effects of Stress Using a Biomonitoring Platform

**DOI:** 10.3390/ijerph15061080

**Published:** 2018-05-26

**Authors:** Susana Rodrigues, Joana S. Paiva, Duarte Dias, Marta Aleixo, Rui Manuel Filipe, João Paulo S. Cunha

**Affiliations:** 1 Institute for Systems Engineering and Computers—Technology and Science (INESC TEC), Porto 4200-465, Portugal; jipaiva@inesctec.pt (J.S.P.); duarte.f.dias@inesctec.pt (D.D.); jpcunha@fe.up.pt (J.P.S.C.); 2 Faculty of Engineering, University of Porto, Porto 4200-465, Portugal; 3 Astronomy and Physics Department, Sciences Faculty, University of Porto, Porto 4169-007, Portugal; 4 Navegação Aérea de Portugal (NAV), EPE, Lisboa 1700-111, Portugal; marta.aleixo@nav.pt (M.A.); rui.filipe@nav.pt (R.M.F.)

**Keywords:** ECG, qOHealth, health monitoring, HRV measures, stress assessment, TSST, reaction times, cognitive performance, air traffic controllers

## Abstract

Stress can impact multiple psychological and physiological human domains. In order to better understand the effect of stress on cognitive performance, and whether this effect is related to an autonomic response to stress, the Trier Social Stress Test (TSST) was used as a testing platform along with a 2-Choice Reaction Time Task. When considering the nature and importance of Air Traffic Controllers (ATCs) work and the fact that they are subjected to high levels of stress, this study was conducted with a sample of ATCs (*n* = 11). Linear Heart Rate Variability (HRV) features were extracted from ATCs electrocardiogram (ECG) acquired using a medical-grade wearable ECG device (Vital Jacket^®^ (1-Lead, Biodevices S.A, Matosinhos, Portugal)). Visual Analogue Scales (VAS) were also used to measure perceived stress. TSST produced statistically significant changes in some HRV parameters (Average of normal-to-normal intervals (AVNN), Standard Deviation of all NN (SDNN), root mean square of differences between successive rhythm-to-rhythm (RR) intervals (RMSSD), pNN20, and LF/HF) and subjective measures of stress, which recovered after the stress task. Although these short-term changes in HRV showed a tendency to normalize, an impairment on cognitive performance was evident. Despite that participant’s reaction times were lower, the accuracy significantly decreased, presenting more errors after performing the acute stress event. Results can also point to the importance of the development of quantified occupational health (qOHealth) devices to allow for the monitoring of stress responses.

## 1. Introduction

Stress could be defined as an imbalance between an excess of demands and the individual ability to cope with them [1]. Stress responses are characterized by an onset of body alterations. Cannon (1914) [2] described these as the “fight-or-flight” response. When a threat is perceived, the autonomic nervous system (ANS) is triggered, the parasympathetic nervous system is suppressed, and the sympathetic nervous system is activated. Consequently, the secretion of stress-related hormones leads to several physiological responses, such as the vasoconstriction of blood vessels, increased blood pressure and breathing rate, increased muscle tension and heart rate (HR), and a decrease in heart rate variability (HRV). Among a variety of physiological indicators (e.g., blood pressure, cortisol, skin conductance, among others), HRV has been proposed as a feasible and reliable method to assess stressful physiological responses [3]. The HRV defines the complex variation of beat-to-beat intervals that are mainly controlled by ANS over the interaction of sympathetic and parasympathetic activity, and it can be indexed by time and frequency-domain parameters [4]. 

Following the guidelines that are presented by the task force of the European Society of Cardiology and the North American Society of Pacing and Electrophysiology [3], different HRV time and spectral domains parameters can be used for the analysis of stress (see Table 1). Methods in the time domain define the intervals between successive normal QRS complexes (the Q-wave, R-wave, and S-wave). Measurements in the frequency domain provide information of how power (variance) distributes as a function of frequency. For time-domain parameters, there are: the Average of NN intervals (AVNN); the Standard Deviation of all NN (SDNN), expressed in milliseconds (ms); the root mean square of differences between successive rhythm-to-rhythm (RR) intervals (RMSSD); and, the percentage of the number of pairs of successive NNs that differ by more than 50 ms as compared to the total number of NN intervals, normally referenced to as pNN50 [3]. For frequency domain analysis, there are the low frequency (LF) component, defined between 0.04–0.15 Hz, as well as the high frequency (HF) component (0.15–0.4 Hz), and their ratio—LF/HF. Castaldo et al. [5] recently conducted a systematic review regarding the associations between acute stress and HRV measures, in order to find the most reliable information about the trends of HRV features during stress (see Table 1). Results suggested that four measures—AVNN, RMSSD, pNN50—in time and non-linear domains, resulted in being significantly depressed during stress and SDNN were also reduced in the majority of studies. The ratio between low and high frequency resulted significantly increased, suggesting a sympathetic activation and a parasympathetic withdrawal during acute stress.

Nevertheless, a vast heterogeneity among studies investigating physiological response to stress was observed in this review, showing that little agreement still exists in the literature for the most suitable HRV-based metric for stress events detection and differentiation [5]. 

Stress can lead to a chronic activation, overload, and ultimately exhaustion of the biological structures (hormonal, cardiovascular, neural, and muscular systems) due to deficient recovery and repair. Long-term consequences include, for example, damages in immune systems [6]. 

As an example, the chronic activation of stress responses, results in the production of glucocorticoid hormones from the endocrine system, and catecholamines from the nervous system, that can alter the function of macrophages and lymphocytes, as well as other cells of the immune system, making the body more vulnerable to diseases, such as infectious illness [7]. Furthermore, prolonged exposure to stress also affects cognition. As an example, McEwen and Sapolsky [8] proposed that chronic stress leads to loss of neurons, particularly in the hippocampus. There also seems to be a relationship between memory and reaction time (RT) and stress also interferes with RT [9]. However, the way stress interferes with RT is not clear. Some studies reflect an improvement on RT that is caused by stress [10], while other studies reflect the contrary [11].

Air Traffic Control (ATC) has been regarded as a very stressful occupation [12]. In fact, economic growth has significantly increased flight demand, which is straining ATC services [13]. According to the International Civil Aviation Organization’s (ICAO) annual global statistics, the total number of passengers that were carried on services rose to 3.8 billion in 2016, which is 6.8 per cent higher than the previous year [14]. When considering the case of Portugal, according to data released by Navegação Aérea de Portugal (NAV, Portugal), the Lisbon total Flight Information Region (FIR) increased 11.2% from 2016 to 2017 [15].

ATC is a very complex process [12,13]. It depends, to a large extent, on human abilities and it encompasses high levels of responsibility not only with regard to risking lives, but also for the high economical costs of aeronautical activities [16]. Much of the Air Traffic Controllers (ATCs) work complexity is characterized by unplanned tasks that require high cognitive load by implying constantly re-plans in response to weather, traffic load management, airport construction, maintenance activities, and other events [17]. The majority of these ATCs tasks are cognitive in nature and are included in highly dynamic and time critical environments. 

The impact of stress in ATCs health has been previously investigated. Accordingly, a study conducted with 30 ATCs and 15 aeronautical information service operators found that stress greatly affected the immune responses of ATCs with more than ten years of experience [18]. Additionally, stress can also interfere with performance [19], and in these professionals in particular, the human factor is considered to be a serious problem in ATC safety [13]. ATCs roles entail a complex set of tasks requiring high levels of knowledge and expertise, as well as the practical use of specific skills concerning cognitive domains, such as: constantly processing changing information, keeping the mental picture of the air traffic situation, keeping and dividing attention among different situations, solving conflicts, planning ahead, working under time pressure, and constantly adapting to changing circumstances [20,21]. Attention is a cognitive ability that has strong implications for the understanding of human performance within ATCs. There is a particular aspect of attention—alertness—that is of great interest for researchers in occupational health area. Alertness is the state of the nervous system that is able to receive and process information at an optimal level [21]. This concept is closely related with the activation theory, particularly with the Yerkes and Dodson law (1908) [22]. This theory provides a relationship between arousal and performance, when considering that every type of behavior requires an optimal degree of arousal that produces maximal performance. In a situation where there is pressure to make a fast response, as can frequently happen in ATC, there will be a tradeoff between the speed of this decision—RT and accuracy [21]. Other important cognitive process is decision making not only for the timely selection of the best decision for a particular situation, but also to assess how that decision will influence the subsequent traffic [23]. It is apparent that the amount of decisions to be made becomes a stressful situation when the controller’s decision-making capacity is struggling to the maximum. This can lead, in case of overload, to a very risky situation that is defined as “loss of picture”. On the other hand, it is frequently reported that many errors often occur during periods of low traffic. This called the attention to the importance of regulate the psycho-physical reactions, maintaining an optimal level of arousal and vigilance, even in situations of “underload” [21].

Despite the importance of investigating the cognitive processes that are involved in ATC, the majority of studies in the literature focused more on ATC tasks complexity and physical workload, rather than analyzing the importance of the cognitive activities of the controller [24,25]. When considering that human error is a major contribution to Air Traffic Management incidents [26], there is a clear need to analyze the cognitive mechanisms by which errors occur and to develop systems that can detect and help to identify the psychophysiological factors that lead to these errors. 

Following the above state of art and considering the importance of investigating the influence of stress on cognitive performance, a controlled experimental protocol was designed for this effect.

First, our goal was to understand whether stress was related to a measurable and quantifiable autonomic response, by identifying the HRV parameters that change significantly under the influence of stress. Our first hypotheses was the following:

**Hypothesis** **1.** *Acute stress would cause a significant reduction in some electrocardiogram (ECG) time-domain parameters—AVNN; SDNN; RMSSD; and, pNN20 and an increase in ECG frequency-domain—LF/HF.*

Cognitive performance was analyzed considering cognitive processes, such as attention and decision making, whereas stimulus–response, which is an important measure in the investigation of cognitive processes among ATCs will be addressed [25]. Therefore, this study also aims to understand the effect of stress on cognitive performance. Our second hypothesis was as follows:

**Hypothesis** **2.** *Acute stress would impair cognitive performance, by increasing the RT and decreasing the accuracy.*

## 2. Materials and Methods

### 2.1. Participants

Eleven Portuguese ATCs (eight males and three females) accepted to participate in this study. This sample represents a total of 3.54% of the overall population of Portuguese ATCs (*N* = 311). The age range was 37 to 54 years (*M* = 46.73 ± 5.90). The years of practice ranged between 13 years to 32 years (*M* = 20.82 ± 6.60). The exclusion criteria for the study were participants having a history of cardiovascular disease and/or taking prescription drugs that are known to affect cardiovascular function. The study was approved by the University of Porto Ethics Committee (29/CEUP/2016). All of the informants were carefully instructed about the study protocol and gave written informed consent prior the examination.

### 2.2. Experimental Setup

**Figure 1:**
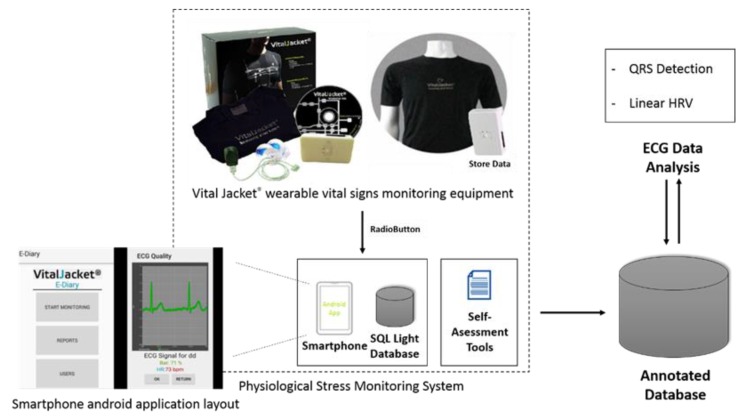
System block diagram explaining physiological monitoring system functioning workflow.

For physiological data acquisition, participants were equipped with Vital Jacket^®^ (1-Lead, Biodevices S.A, Matosinhos, Portugal) [27,28] (Figure 1). The Vital Jacket^®^ is a wearable bio-monitoring platform (in form of a t-shirt) that is able to collect medical-grade ECG signals and actigraphy in real-time. This equipment is certified according to the MDD93/42/EEC medical device directive holding the European Conformity medical device mark [29].

This system includes an Android application that pairs with Vital Jacket^®^ via Bluetooth and enables the exact annotation of events in the ECG trace, using “Radiobuttons”. The android application stores all of the information about the events in an SQL Light DataBase, being this information synchronized with the ECG trace. All of these synchronized data are then exported to a database, creating an annotated database. Then, the data can be used for processing and analysis (QRS detection and Linear HRV), always ensuring the synchronization between the events information and the data collected by the Vital Jacket^®^ (see Figure 1). 

In order to collect psychological stress data, the Spielberg State-Trait Anxiety Inventory (STAI six-item short-form) was used [30]. This self-report allows to evaluate emotional, physical, and cognitive aspects of stress. When considering that our goal was to understand the differences in state anxiety from the beginning to the end of the protocol we chose the short-form of the STAI that, according to the authors, is more sensitive to fluctuations in state anxiety [30].

Additionally, Visual Analogue Scales (VAS) were used to assess perceived stress along the protocol [31]. This measure required participants to rate the average level of perceived stress that is experienced during the tasks by marking any point on a 10-cm line ranging from “None” to “As bad as it could be”.

Demographic and medical surveys were also used in order to assess participant’s current health state and assure that the inclusion criteria were fulfilled.

Cognitive performance was assessed using a 2-Choice Reaction Time Task based on the study of Weissman et al. [32]. This is a global/local selective-attention task, whereas participants identified either the large, global letter or the small, local letters of a hierarchically organized visual object. Their response time and correct/incorrect/missed answers were recorded. 

### 2.3. Procedure

**Figure 2:**
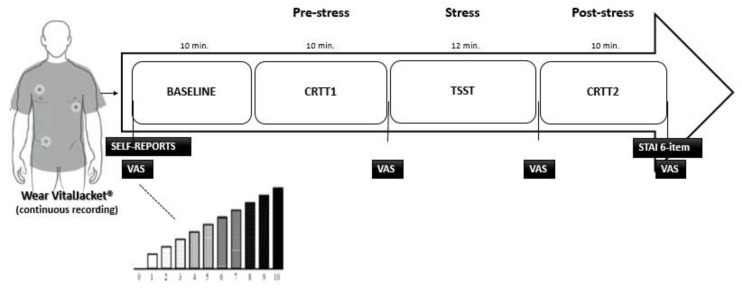
Diagram of the protocol. VAS—Visual Analogue Scales. CRTT—Choice Reaction Time Task. TSST—Trier Social Stress Test. Self-reports—include demographic, medical survey and State-Trait Anxiety Inventory (STAI) six-item.

A presentation session was organized to explain the aim and the overall protocol of the study. The protocol lasted approximately 45 min and included a combination of tasks, as shown in Figure 2. 

**Figure 3:**
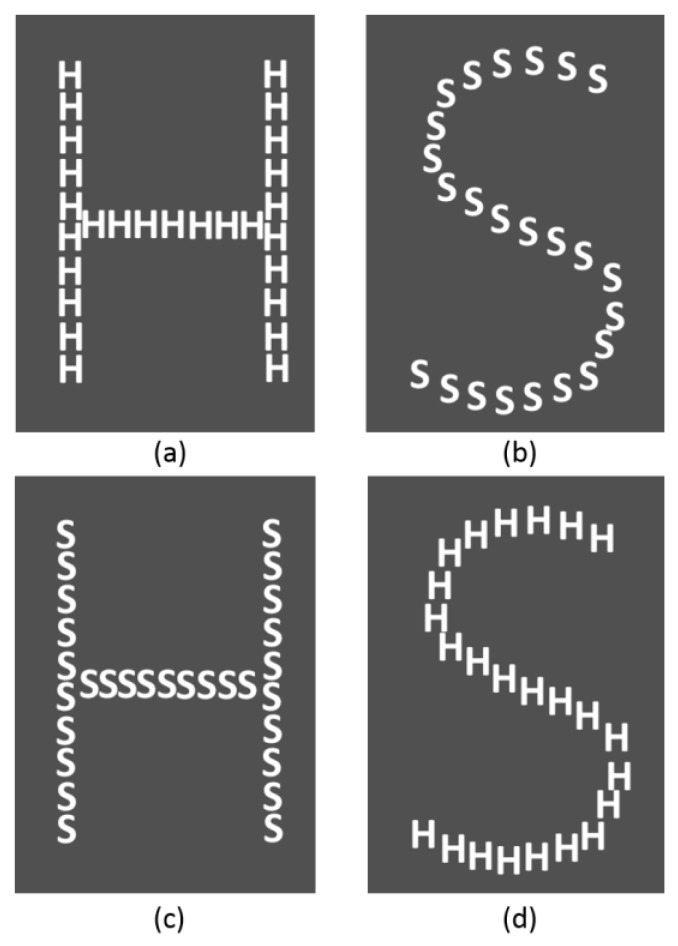
The four types of stimuli used in the CRTT. (**a**,**b**) Congruent stimuli. (**c**,**d**) Incongruent stimuli.

**Figure 4:**
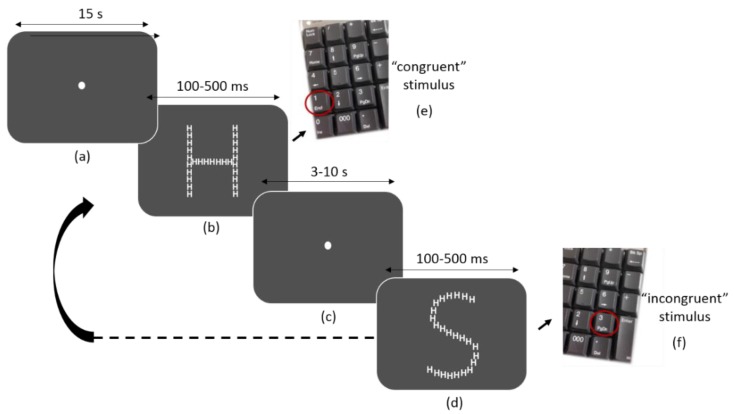
The four types of stimuli used in the CRTT. (**a**,**b**) Congruent stimuli. (**c**,**d**) Incongruent stimuli.

Participants completed the demographic, medical survey, and STAI six-item short-form. Then, they were equipped with Vital Jacket^®^ and they were allowed to sit comfortably for a 10-min resting baseline. Additionally, at the beginning of the protocol and at the end of each task, ATCs were requested to fill in the VAS in order to assess perceived stress. Afterwards, they started by performing a 10 min custom-made 2-Choice Reaction Time Task (CRTT) while sitting in a comfortable chair [32]. The stimuli sequence file used in this task was developed in Matlab (R2015a, The MathWorks, Natick, MA, USA) using the Psychophysics Toolbox for display. Participants were requested to press a button as quickly and as accurately as possible to indicate the identity (H or S) of the large, global letter or the small, local letters of a hierarchically organized visual stimulus (for example, a large H made up of small Ss). In half the trials, the global and local letters were mapped to the same response (congruent trials; for example, a large H made of small Hs); in the other half, they were mapped to different responses (incongruent trials; for example, a large H made of small Ss) (Figure 3). A white fixation dot was displayed at the center of the screen for approximately 15 s in the beginning of main run—Figure 4. (a). Thereafter, one of the four possible stimuli (see Figure 3) was randomly chosen and presented with a interstimuli interval between 3 and 10 s to avoid expectancy effects. Participants were asked to press the key ‘1’ on a keyboard for the “congruent” stimulus—Figure 3a,b—whereas it was expected that subjects pressed the key ‘3’ in response to the “incongruent” one—Figure 3c,d. Targets were therefore shown also for a random period of time between 100 and 500 ms. The white fixation dot was presented in the center of the screen during the interstimuli interval and subjects were asked to continue fixating the gaze on it until a new stimulus was presented—Figure 4c.

This task was performed twice, at the beginning of the protocol (CRTT 1; pre-stress condition) and after the stress task (CRTT 2; post-stress condition). 

The stress procedure was based on an acute psychosocial stress paradigm, the Trier Social Stress Test (TSST) [33]. This task was chosen when considering its components of unpredictability and uncontrollability, as a means to represent those elements of stressors that are encountered daily by ATCs. 

In short, in the TSST, the participants were asked to deliver a speech for 3–5 min and to perform an arithmetic task (e.g., count down from 1022 by 13’s for two minutes). When making a mistake, the participants were asked to start over in front of an evaluating committee. The committee consisted of three jury members that do not respond emotionally during the test, which makes the situation stressful for the participant. It is important to note that the arithmetic procedure was reduced from five to two minutes. This change from the original version was made, firstly, because it was not also our goal to perform an individual analysis of each period of the TSST. Secondly, we considered in the ECG statistical data analysis a balanced number of 5-min of ECG blocks in each moment. Hence, the minimal number of 5-min ECG blocks was two. Therefore, we just need 10 min of ECG data in the stress condition. Although the TSST can been modified to meet the needs of research groups, its general structure was respected [34].

During all session, one of the researchers used the android smartphone application to mark the events that synchronized with the ECG. At the end of the protocol, participants filled in STAI six-item short-form again.

### 2.4. Data Analysis

In order to test Hypothesis 1 (Acute stress would cause a significant reduction in some ECG time-domain parameters—AVNN; SDNN; RMSSD; and, pNN20 and an increase in ECG frequency-domain—LF/HF) an ECG data analysis was performed. In order to extract heartbeat information from the ECG recordings was used a software from Biodevices S.A (Matosinhos, Portugal), with an ECG analyzer. This software has an algorithm based on the one that was developed by Pan Tompkins [35] incorporating ECG physiological filters to detect the “R” points of the ECG waveform. This analyzer was used to extract the RR interval (time between two consecutives “R” peaks in the ECG). A simple verification according to Clifford et al. [36] was made to verify if all the RR intervals were physiologically correct. Hence, all of the RR intervals lower than 300 ms and greater than 3000 ms were removed. Additionally, all of the RR intervals that changed by more than 400 ms with respect to the previous VALID RR interval and all RR intervals that change by more than 25% with respect to the mean of the five last VALID RR intervals were removed. The RR intervals that have physiological acceptance are named normal-to-normal (NN) intervals. Hence, following the guidelines that are presented by the task force of the European Society of Cardiology and the North American Society of Pacing and Electrophysiology [3], different HRV time and spectral domains parameters were used in this study: AVNN; SDNN; RMSSD; pNN20; and, LF/HF. For pNNx, a value of 50 ms is the most commonly used. However, there has been evidence that shorter durations of pNNx can help to improve the analysis of HRV changes [37]. Hence, we used pNN20, instead of pNN50, due to the interesting results that were obtained by Schaaff et al. [38].

The second hypothesis (acute stress would impair cognitive performance, by increasing the RT and decreasing the accuracy) was tested while considering the median Reaction Time (RT) and the Accuracy (number of correct answers/answered responses) when performing CRTT. Z-score values were considered regarding cognitive performance group statistical analysis.

All data were statistical analyzed using IBM SPSS AMOS (v.24) software (IBM Corp., Armonk, New York). Taking into account the few number of population samples, some parameters failed in the normality test, so all parameters were analyzed using non-parametric statistical tests. Wilcoxon Signed Rank Test and the Friedman Test were the nonparametric alternatives used to compare averages between study time points. Paired-samples *t*-test was used as post-hoc pairwise comparisons to determine the exact differences between the time pairs [39]. Spearman correlation was performed in order to find significant correlations between the cognitive performance measures (RT and Accuracy). Median reaction time (RT) values were considered instead of mean values, because RTs are not normally distributed with a longer tail of slow when compared with fast responses [40]. 

## 3. Results

### 3.1. Psychological Stress Scores

**Figure 5:**
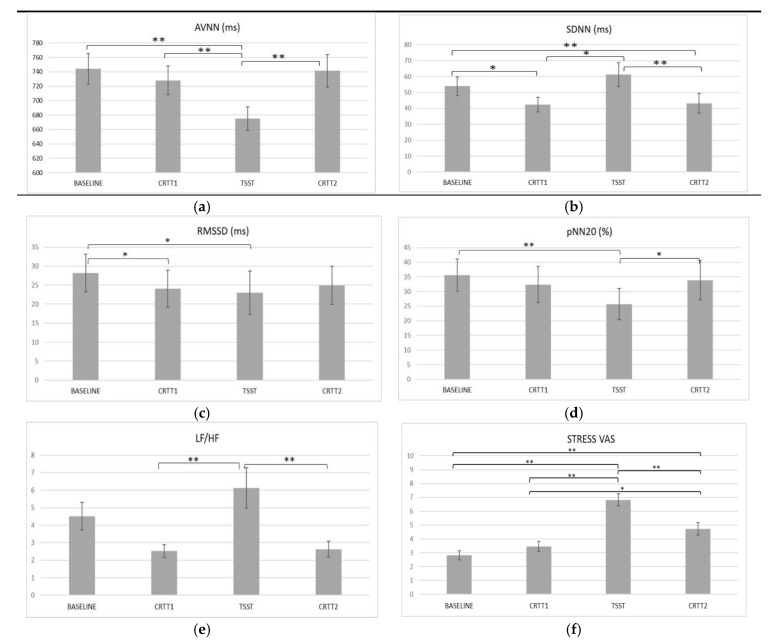
Mean statistical significant differences for electrocardiogram (ECG)-derived measures—(**a**) Average of normal-to-normal intervals (AVNN); (**b**) Standard Deviation of all NN (SDNN); (**c**) root mean square of differences between successive rhythm-to-rhythm (RR) intervals (RMSSD); (**d**) pNN20; (**e**) LF/HF and subjective stress measures—and, (**f**) VAS across study time points (*p* < 0.01; Friedman test). * *p* < 0.05; ** *p* < 0.01; *** *p* < 0.001 for post-hoc pairwise comparisons.

To test the Hypothesis 1, stress perceptions were firstly analyzed to understand if the acute stress task was self-perceived as stressful. Wilcoxon Signed Rank Test was used to analyze STAI six-item short-form data. Results revealed a statistically significant increase of stress perceptions, *p* < 0.05. The median score on STAI six-item short-form increased from the beginning of the protocol (*Md* = 1.5) to the end of the protocol (*Md* = 2.0). The Friedman Test was used to analyze VAS scores along the protocol. Results indicated that there was a statistically significant change in the perceived stress between the four time points of the study: baseline, CRTT1, TSST, and CRTT2—*p* < 0.001. Post-hoc pairwise comparisons showed that perceived stress significantly increased from baseline (*M* = 2.82) to TSST (*M* = 6.82) and to CRTT2 (*M* = 4.73) and from CRTT1 (*M* = 3.45) to TSST (*M* = 6.82) and to CRTT2 (*M* = 4.73). Finally, VAS significantly decreased from TSST (*M* = 6.82) to CRTT2 (*M* = 4.73) (Figure 5f). The highest mean value that was obtained for VAS was after TSST (*M* = 6.82). These results support the idea that the TSST was a stress-inducing task. 

### 3.2. ECG Data 

To test the Hypothesis 1, regarding the physiological impact of acute stress, HRV-based metrics of baseline ECG that were collected at the 10 first minutes of the protocol were compared with HRV measures of three important time points of the study: the pre-stress condition—CRTT1, the stress condition—TSST; and, the post-stress condition—CRTT2. The time domain HRV features AVNN, SDNN, RMSSD, pNN20, and frequency domain LF/HF were used in these analyses. Friedman Test was used to understand if there were statistically significant differences in these parameters across the four time points of the study (Figure 5). 

Regarding AVNN, significant differences were found between the four time points, *p* < 0.001. Post-hoc pairwise comparisons showed that AVNN significantly decrease from baseline (*M* = 744.15 ms) to TSST (*M* = 675.23 ms). A significant decrease from CRTT1 (*M* = 727.95 ms) to TSST (*M* = 675.23 ms) was also found. Finally, an increase was observed from TSST (*M* = 675.23 ms) to CRTT2 (*M* = 741.37 ms). Inspection of the mean values showed that AVNN achieved the lowest value during TSST (*M* = 675.23 ms) (Figure 5a).

Regarding the SDNN, *p* < 0.01, post-hoc pairwise comparisons showed that SDNN significantly decreased from baseline (*M* = 53.98 ms) to CRTT1 (*M* = 42.36 ms) and from baseline to CRTT2 (*M* = 43.21 ms). Additionally, a significantly increase was found between CRTT1 (*M* = 42.36 ms) and TSST (*M* = 61.20 ms), then decreasing after CRRT2 (*M* = 43.21 ms). Inspection of the mean values showed that SDNN achieved the highest value during TSST (*M* = 61.50 ms) (Figure 5b). 

For RMSSD, *p* < 0.01, post-hoc pairwise comparisons showed that RMSSD significantly decreased from baseline (*M* = 28.22 ms) to CRTT1 (*M* = 24.06 ms) and to TSST (*M* = 23.03 ms). The lowest value was obtained during TSST (*M* = 23.03 ms). (Figure 5c). 

Regarding pNN20, *p* < 0.01, post-hoc pairwise comparisons revealed that pNN20 significantly decreased from baseline (*M* = 35.61%) to TSST (*M* = 25.70%), and it then increased from TSST to CRTT2 (*M* = 33.84%). Inspection of the mean values showed that pNN20 achieved the highest value during TSST (*M* = 25.70%) (Figure 5d).

Finally, for LF/HF, *p* < 0.01, post-hoc pairwise comparisons showed that LF/HF significantly increased from CRTT1 (*M* = 2.52), to TSST (*M* = 6.13), then decreasing to CRTT2 (*M* = 2.63). The highest value was obtained during TSST (*M* = 6.13) (Figure 5e). With the exception of SDNN, these results provide support for Hypotheses 1.

### 3.3. Cognitive Performance

**Figure 6:**
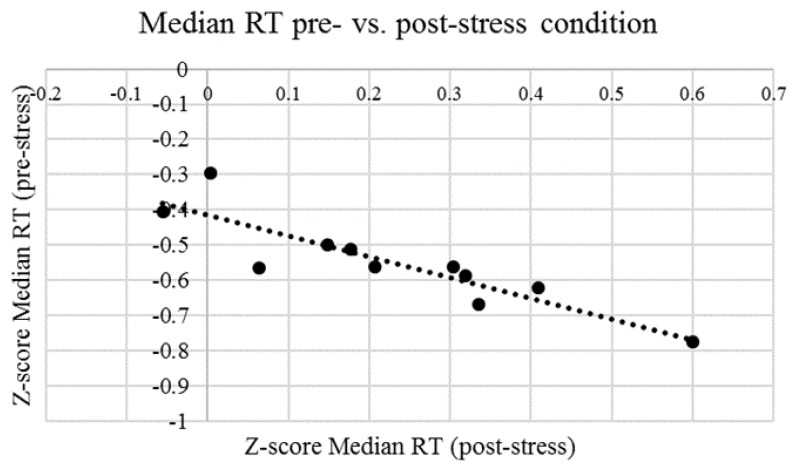
Graphic representing the relation between Z-score Median reaction time (RT) pre- (CRTT1) and post-stress (CRTT2) conditions and corresponding fit line. Each dot point in the graphic represents each subject.

To test Hypothesis 2, the Wilcoxon test was performed and significant differences were obtained for Median RT and Accuracy levels. Results revealed a statistically significant decrease of RTs from CRTT1 to CRTT2, *p* < 0.01. The median Z-score on RTs decreased from CRTT1 (*Md* = 0.21) to CRTT2 (*Md* = −0.56). Accuracy levels also change significantly, *p* < 0.05. The median score on accuracy significantly decreased from CRTT1 (*Md* = 0.95) to CRTT2 (*Md* = 0.88). Additionally, a negative and strong correlation was found between the RTs that were obtained in CRTT1 and CRTT2 (*r* = −0.89, *p* < 0.001) (Figure 6). These results partially support the Hypothesis 2, when considering that RTs decreased after the acute stress task.

## 4. Discussion

This study examined the effects of acute stress on cognitive performance and whether this effect was related to the autonomic response to stress, by analyzing several HRV parameters, in order to identify those that change significantly under the influence of stress. The TSST was developed as a gold standard procedure that is designed to produce a psychophysiological stress response [34,41].

Our first hypothesis stated that acute stress would have an impact on physiology, particularly a reduction in some ECG time-domains—AVNN, SDNN, RMSSD, and pNN20, and an increase in ECG frequency-domains—LF/HF. In order to test this hypothesis, we first analyze psychological data, in order to verify if the stress task was in fact stressful for the participants, as suggested by Lazarus and Folkman, when defining stress [1]. 

Psychological stress results, based on VAS, along with some HRV parameters showed that the chosen stress paradigm indeed produced stress and was thus suited for the aim of this study. Therefore, it showed that short-term psychological stress caused significant changes in time domain measures (AVNN, SDNN, RMSSD, pNN20), as well as in frequency domain parameters (LH/HF) between the main time points of the study.

Lower values found for AVNN during the stress condition suggest an overall increase in heart rate or cardiac sympatho-excitation, typical in stress situations [42]. This response suggests a higher degree of activation of the cardiovascular system to compensate the body physiological responsiveness to stress. Hence, lower values are associated with stress responses [6].

Regarding SDNN, there were significant changes from CRTT1 to TSST and to CRTT2, whereas this value achieved higher values during the stress condition. This is an unexpected result, because acute stress tends to cause a decrease in SDNN [5]. However, these findings are similar to those that were obtained by Schubert et al. [43] in a study examining the stress effects on HRV in 50 healthy subjects using a short-term stressor reactivity that was assessed with a speech task. Results suggested that SDNN increased during the stress condition. The authors proposed that talking loud, and particularly, doing stressful mental arithmetic’s, were associated with a decrease in respiratory rate and a relative reduction in ventilation, which resulted in an increase in SDNN. Such evidence on the influence of speech-related respiratory patterns on linear HRV measures could explain why in the current study the stress condition (TSST—that also includes a speech task and an arithmetic task) was associated with decreases in SDNN, independently of changes in parasympathetic modulation of heart rate. 

Changes in the RMSSD and pNN20 are both reported in the literature to reflect high frequency changes in heart rate, and therefore parasympathetic modulation [3]. Hence, decreases of AVNN, RMSSD, and pNN20 during the stress condition reflected a depressed HRV, which is typical during stress [5]. Within the frequency-domain, a significant increase in the LF/HF ratio during the stress condition was also found, indicating the marked effect of stress on sympathovagal balance. Higher values reflect the domination of the sympathetic system over the parasympathetic one, which is typical of stress responses [44]. These results were concordant with an elevation of self-perception measures of stress (VAS) in the stress condition. Additionally, STAI 6-item results also suggested an increment on self-perception of stress, from the beginning of the protocol to the end. These results confirm our hypothesis 1, with the exception of SDNN that increased with stress. 

Our second hypothesis stated that acute stress would impair cognitive performance, by increasing the RTs and decreasing the accuracy. For the analysis of the impact of stress on cognitive performance, an RT Task was chosen to require cognitive skills, such as attention and decision making, under the pressure instruction to make faster responses. Results partially confirm our hypothesis because it was found that after the stress condition, the participants responded faster, transducing into decreased RTs, however presenting more errors—decreased accuracy. Errors among these professionals might lead to critical events when considering all of the dimensions involved [26]. This issue was already discussed in ATC situations, while considering that conditions that increase arousal levels, lead to faster, but often less accurate, responding [21]. In certain ATC situations, where any delay could cause negative consequences, the pressure to make faster responses, sometimes without sufficient information can lead to errors. These results, which are in line with Yerkes and Dodson law (1908) [22], reinforce the need to find optimal stress levels for maximal performance in terms of both productivity levels and safety. It is important to bear in mind that this study did not represent ATCs real world conditions, and they were exposed to a different stressor. As the role of human factors is more and more important for air traffic safety, the research on the controller RT in different simulated air traffic situations and real work environments needs further development [25].

It was also found in the current study that, despite the fact that after the stress condition, and during the CRTT2, HRV parameters showed a tendency to “normalize”, participants presented an impaired cognitive performance, which affected their attention and decision making ability. Accordingly, in a study that was conducted with 23 healthy participants aiming to investigate the effects of TSST on impaired attention and working memory, it was concluded that abnormalities in attention persisted for at least 30 min following the stressor [41]. These results enhance even more the negative impact of stress levels on cognitive abilities in short and long terms. These findings start to clarify our understanding of some of the maladaptive behaviors that were observed in response to exposure to stressful events. Applied implications for ergonomic work could include, for example, routinely health and stress levels analysis, to implement forward-rotating shift systems, and breaks in line with their position’s workload that can help them how to better cope with the daily work stress. 

This study is not without limitations. Particularly, the reduced sample size and the fact that cognitive performance was analyzed using only one simple cognitive task. Nevertheless, this study was experimental and it was the first part of a bigger study that includes an ATC simulator task assessment and an ambulatory protocol that was developed during real ATC situations. 

## 5. Conclusions

This study showed that TSST is a successful testing platform for employing technological advances in the assessment of physiology. In this particular case, some HRV metrics (AVNN, SDNN, RMSSD, pNN20, and LF/HF) significantly change under the influence of acute stress. Additionally, stress impaired cognitive performance, because, despite participant’s reaction times were lower, the accuracy significantly decreased, presenting more errors after performing the acute stress event. 

The current study is also pioneer in terms of the specific population under study and the methodology that was used, providing an important insight into the direct effects of stress on health and performance. This allows for future avenues for new perspectives on stress-related diseases, and the development of personalized and quantified occupational health (qOHealth) devices allowing for real life monitoring of stress responses. 

## 6. Patents

Cunha, J.P.S.; Paiva, J.S. Method and device for detecting stress using beat-to-beat ECG features. Patent request NR. EP 17188217.8 (August 2017).

## Figures and Tables

**Table 1:** Descriptions of the heart rate variability (HRV) measures analyzed [3] and their trend under stress [5].

Domain	Measure	Description	Features Trend under Stress
Time-domain	AVNN	Average of NN intervals (ms)	↓
	SDNN	Standard Deviation of all NN intervals (ms)	↓↑
	RMSSD	Root mean square of differences of successive NN intervals (ms)	↓
	pNN50	NN variations above 50 ms (%)	↓
Frequency-domain	LF/HF	Ratio of Low Frequency and High Frequency power band	↑
